# The impact of physical training on length of hospital stay and physical function in patients hospitalized with community-acquired pneumonia: protocol for a randomized controlled trial

**DOI:** 10.1186/s13063-021-05503-2

**Published:** 2021-08-28

**Authors:** Camilla Koch Ryrsø, Daniel Faurholt-Jepsen, Christian Ritz, Bente Klarlund Pedersen, Maria Hein Hegelund, Arnold Matovu Dungu, Adin Sejdic, Birgitte Lindegaard, Rikke Krogh-Madsen

**Affiliations:** 1 Department of Pulmonary and Infectious Diseases, Nordsjællands Hospital, University of Copenhagen, Hillerød, Denmark; 2 Centre for Physical Activity Research, Rigshospitalet, University of Copenhagen, Copenhagen, Denmark; 3 Department of Infectious Diseases, Copenhagen University Hospital, Rigshospitalet, Copenhagen, Denmark; 4 Department of Nutrition, Exercise and Sports, University of Copenhagen, Copenhagen, Denmark; 5 Department of Clinical Medicine, University of Copenhagen, Copenhagen, Denmark; 6 Department of Infectious Diseases, Copenhagen University Hospital, Hvidovre, Copenhagen, Denmark

**Keywords:** Community-acquired pneumonia, Physical training, Length of hospital stay, Lean mass, Functional ability

## Abstract

**Background:**

Community-acquired pneumonia (CAP) is a leading cause of hospitalization worldwide. Bed rest with low levels of physical activity is common during periods of hospitalization and leads to functional decline as well as increased risk of complications. The aim of this study is to assess the effect of supervised physical training during hospitalization with CAP compared with standard usual care for CAP on length of hospital stay, risk of readmission, mortality risk, physical capacity, muscle and fat mass, muscle strength, metabolic function, systemic inflammation, health-related quality of life, and physical activity level.

**Methods:**

This study is a randomized controlled trial with three parallel experimental arms. Based on a sample size calculation, a total of 210 patients admitted with CAP at Nordsjællands Hospital, Hillerød, Denmark, will be recruited. Patients will be randomly allocated (1:1:1) to either (1) standard usual care, (2) standard usual care combined with in-bed cycling, or (3) standard usual care combined with exercises from a booklet. The primary outcome is differences in length of hospital stay between groups, with secondary outcomes being differences between groups in time to (1) 90-day readmission and (2) 180-day mortality. Further secondary outcomes are differences in changes from baseline between groups in (3) lean mass, (4) fat mass, (5) fat-free mass, (6) physical capacity, (7) health-related quality of life, (8) systemic inflammation, and (9) physical activity level after discharge. Data on length of hospital stay, readmission, and mortality will be collected from patient files, while total lean, fat, and fat-free mass will be quantitated by dual-energy x-ray absorptiometry and bioelectrical impedance analysis. Physical function will be assessed using grip strength, 30-s chair stand tests, and Barthel Index-100. Health-related quality of life will be assessed with the EQ-5D-5L questionnaire. Systemic inflammation will be assessed in blood samples, while accelerometers are used for measuring physical activity.

**Discussion:**

If a simple physical training program appears to diminish the impact of critical illness and hospitalization on clinical outcome, mobility, and health-related quality of life, it may lead to novel therapeutic approaches in the treatment of patients hospitalized with CAP.

**Trial registration:**

ClinicalTrials.gov NCT04094636. Registered on 1 April 2019

**Supplementary Information:**

The online version contains supplementary material available at 10.1186/s13063-021-05503-2.

## Background

**Fig. 1:**
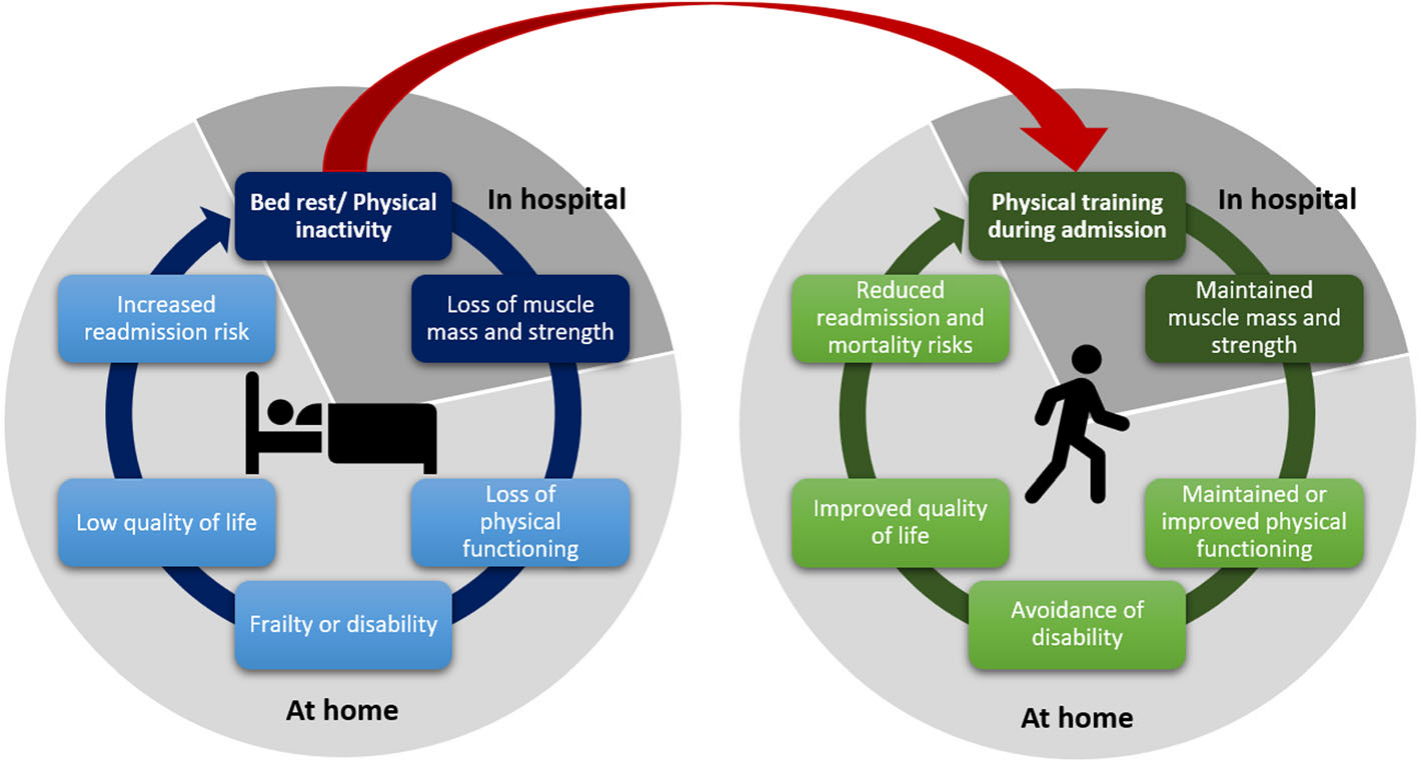
Breaking the vicious cycle of hospitalization, disability, and poor prognosis through physical training. Dark colors, e.g., dark gray, dark blue, and dark green, indicate processes which takes place in hospital during hospitalization. Lighter colors, e.g., light gray, light blue, and light green, indicate time periods, which takes place at home after discharge

Community-acquired pneumonia (CAP) is a leading cause of hospitalization and death from infectious diseases worldwide [1], with an overall mortality of 14% in patients hospitalized with CAP [2]. The incidence of patients hospitalized with pneumonia has been increasing in Denmark through the past 2 decades from 288 to 809 per 100.000 person-years and is predicted to continue rising due to the aging of the population [3, 4]. One out of five patients are readmitted within 30 days, making readmissions a significant burden both for the patient and the health care system [5]. Hospitalization is associated with bed rest which can result in prolonged physical inactivity and immobilization. In fact, patients hospitalized with CAP spent over 90% of their waking hours being physically inactive, i.e., either laying or sitting down, with only 926 steps per day during admission [6, 7]. Moderate to severe frailty is a predictor of lower daily step count. It has been reported that frail patients take 59% less steps per day during admission compared to robust patients [6], which can exacerbate physical deterioration several weeks after discharge [8]. Frail patients with frequent readmissions may be prone to a more rapid decline in functional ability and associated deteriorations in health-related quality of life [9], which may cause a *viscous cycle* of physical inactivity with negative effect on the prognosis [10–12] (Fig. 1).

Previous studies have shown that only 10–14 days of bed rest leads to a substantial loss of lower extremity muscle mass and muscle strength, increase in visceral fat, and impairments in insulin sensitivity in healthy individuals [13–16]. Moreover, these bed rest-related changes were associated with a reduction in physical activity level during the week following bed rest [13]. However, early mobilization of patients with CAP within the first 24 h of hospitalization has been shown to decrease length of hospital stay by 1.1 to 2.1 days without increasing the risk of adverse events [17–19]. In addition, for every increase in the daily step count above 500 steps, length of hospital stay was reduced by 11% among patients with CAP [6]. Moreover, we have previously shown that initiating supervised early pulmonary rehabilitation of patients with acute exacerbation of chronic obstructive pulmonary disease (COPD) during hospitalization is superior to standard usual care in terms of improving prognosis (reduces the risk of COPD-related hospital readmissions), health-related quality of life, and walking distance [20].

Even though international guidelines for the management of patients hospitalized with CAP recommend early mobilization as part of standard of care for CAP [21], the physical activity level among patients hospitalized with CAP remains low. Despite the emerging evidence that physical activity during hospitalization for respiratory conditions is beneficial in terms of improving functional outcomes, it is not consistently prioritized in clinical practice. Therefore, interventions that increases physical activity and interrupt sedentary time during hospitalization are crucial (Fig. 1).

## Methods/design

### Study design and study setting

**Fig. 2:**
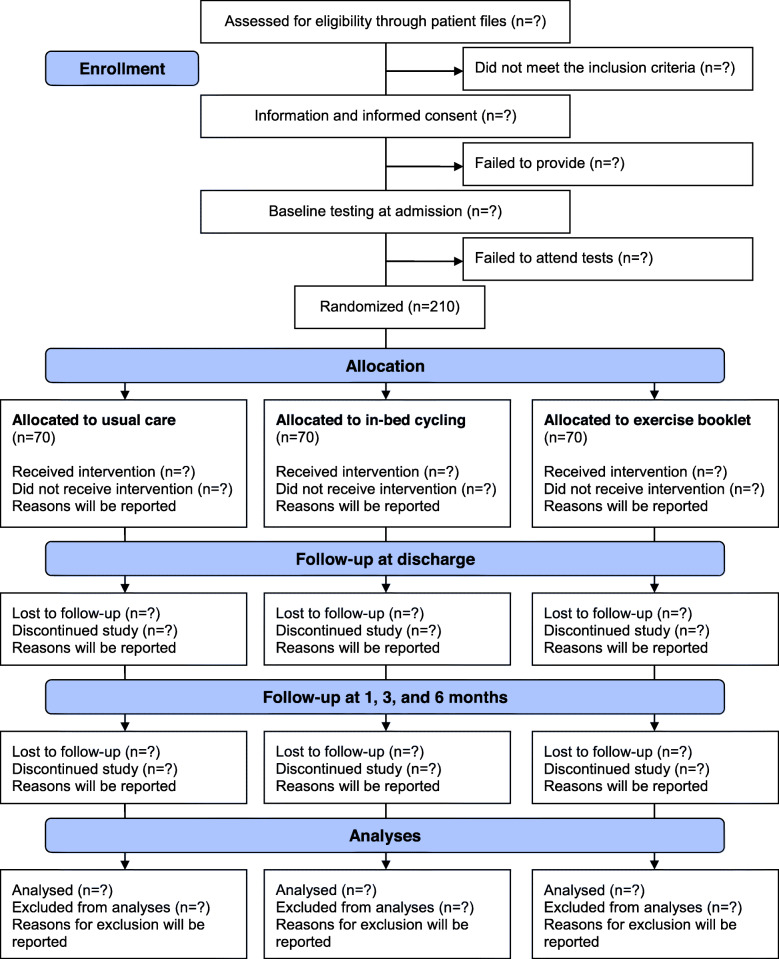
Flow of patients though the study. Any reasons for discontinuation or exclusion from analysis are reported

The study is a single-center, randomized, superiority, open-label, controlled trial with three parallel experimental arms, nested in a prospective cohort study. Over a 3-year period, all patients hospitalized with suspicion of lower respiratory tract infection at the Emergency Department at Nordsjællands Hospital, Hillerød, Denmark, will be screened for eligibility. Based on a sample size calculation, a total of 210 patients hospitalized with CAP are randomized in a ratio of 1:1:1 to standard usual care or standard usual care combined with daily supervised physical training consisting of either in-bed cycling or booklet exercises as depicted in Fig. 2. The enrolment period began on 1 April 2019 and is estimated to be completed by 1 March 2022. All baseline and discharge tests are carried out at the Department of Pulmonary and Infectious Diseases, Nordsjællands Hospital, Hillerød, Denmark. The follow-up tests are carried out at Department of Pulmonary and Infectious Diseases, Nordsjællands Hospital, Hillerød, Denmark or as home visits.

### Study hypotheses and aims

We hypothesize that standard usual care combined with daily supervised in-bed cycling or standard usual care combined with booklet exercises is superior to standard usual care alone in reducing length of hospital stay, reducing the risk of 90-day readmission, and 180-day mortality, counteracting loss of muscle mass, muscle strength, functional ability, low/reduced health-related quality of life, increase in fat mass, reducing systemic inflammation, and increasing the physical activity level after discharge.

The primary aim of the study is to determine if supervised in-bed cycling or supervised booklet exercises during hospitalization with CAP reduces length of hospital stay. Secondary aims are to determine if supervised in-bed cycling or supervised booklet exercises during hospitalization with CAP improves prognosis (e.g., reduces the risk of readmission and mortality) and minimizes/counteracts hospital-related changes in body composition, muscle strength, functional ability, health-related quality of life, systemic inflammation, and physical activity level after discharge. Additional aims are to investigate if supervised in-bed cycling or supervised booklet exercises during hospitalization with CAP improves glucose metabolism, diabetes and health status, and frailty.

## Participant selection

### Eligibility

Patients are screened through patient files for eligibility within the first 24 h after hospital admission by a physician or study nurse affiliated with the study. Patients are eligible if they are age ≥18 years and hospitalized with CAP. The definition of pneumonia is presence of a new infiltrate on chest X-ray/CT and minimum one of the following symptoms: temperature < 35 °C or ≥38 °C, cough, pleuritic chest pain, dyspnea, or focal chest signs on auscultation. To be enrolled, patients must have an expected length of hospitalization of ≥72 h and the ability to move their legs. Patients will be excluded if they are immunosuppressed (cancer chemotherapy ≤28 days, neutropenia ≤1000 cells/μL, ≥20 mg prednisolone-equivalent/day >14 days or other immunosuppressive drugs, HIV infection, former transplant) or have been hospitalized within ≤14 days (Table 1).

**Table 1:** Eligibility of study participants

Inclusion criteria	Exclusion criteria
1. Age ≥ 18 years 2. Expected length of hospitalization ≥72 h 3. New infiltrate on chest X-ray/CT 4. One or more of the following symptoms: a. Temperature <35 °C or ≥38 °C b. Cough c. Dyspnea d. Pleuritic chest pain e. Focal chest signs on auscultation	1. Patients unable to give written consent 2. Hospitalization within ≤ 4 days 3. Inability to move their legs 4. Immunosuppression a. Treatment with ≥20 mg corticosteroids >2 weeks b. Chemotherapy c. Neutropenia with neutrophils <0.5 × 10^9^/L d. Ongoing treatment with biological drugs e. Chronic HIV-infection with CD4 cell count <350 mio/L f. Immunosuppression after organ transplantation 5. Terminal ill patients where active treatment is stopped within the first 48 h of admission

## Interventions

The intervention is a combination of standard usual care for CAP and daily supervised physical training tested with 2 different training modalities: exercise booklet and in-bed cycling. The resulting intervention and standard usual care group are depicted in Fig. 2. While mobilization defined as movement out of bed with change from horizontal to upright position for at least 20 min per day is imbedded in the standardized treatment for patients hospitalized with CAP [21], it is often neglected and only sporadically addressed. Own data shows that only 21% of the patients have notes on mobilization in their patient file. The lack of systematic mobilization is criticizable as the impact on patient-related outcomes is well-established [17, 22]. Barriers for mobilization often includes restricted/lack of time for mobilization, patient’s lack of motivation for mobilization, and problems with nurses not viewing mobilization as part of their core task [23]. In Denmark, hospitalized patients only receive physiotherapy if it is prescribed by the physician. The study will record from patient files if a patient receives respiratory physiotherapy, exercise training by a physiotherapist, mobilization, or rehabilitation plans.

### Exercise booklet

**Fig. 3:**
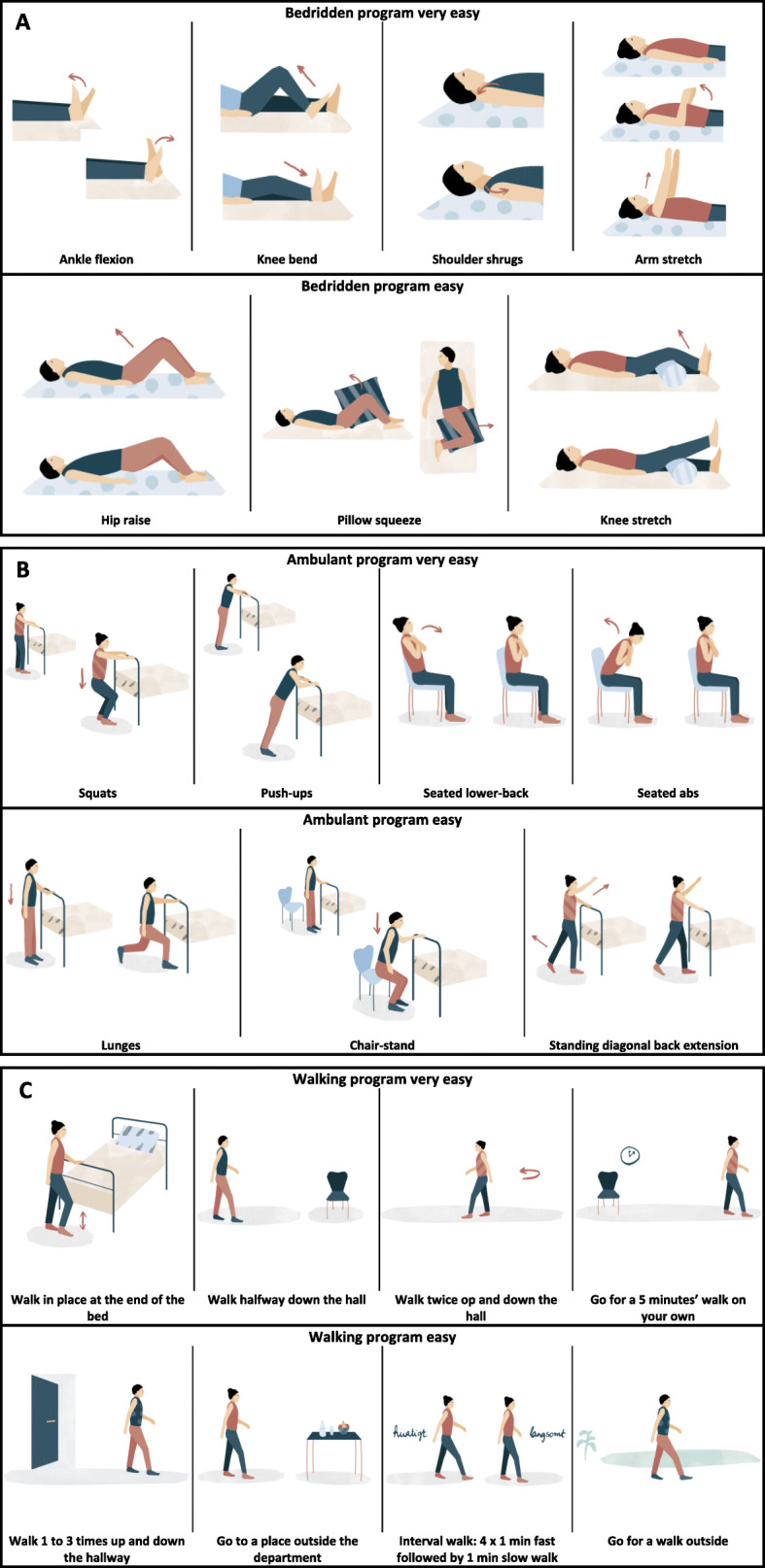
Exercise programs from the exercise booklet. The exercises are designed for bedridden and ambulant patients. The exercises are divided into 3 programs; **A**, **B**, and **C**, respectively. Programs **A** and **B** include strengthening exercises divided into varying degrees of difficulty (e.g., very easy or easy) for both bedridden (**A**) and ambulant patients (**B**). Program C includes different walking programs

The exercise intervention with the exercise booklet is chosen as it is aims towards the sick hospitalized patient. The exercise booklet (Fig. 3) includes strengthening and walking exercises with varying degree of difficulty that can be easily done during hospitalization to ensure that all patients are challenged to be physically active, irrespectively of mobility (bedridden or ambulant). The functional exercises from the booklet are designed to challenge stability, strength, and endurance in order to train the patient’s ability to perform activities of everyday life after discharge. Patients allocated to the exercise booklet group will daily receive 30 min of supervised physical training after an exercise booklet (entitled “Syg men Sund og Aktiv” [“Sick but Healthy and Active” in English], The Centre for Physical Activity Research, Copenhagen, Denmark). Every exercise session is supervised and instruction by an exercise physiologist or physiotherapist student. The selection of exercises is left to the exercise instructor training the patient. The patients will perform 3 times 10 repetitions of each selected exercise from the strengthening programs for either bedridden (Fig. 3a) or ambulant (Fig. 3b) patients. The strengthening exercises will be combined with exercises from the walking program (Fig. 3c) giving each exercise session a total of 30 min.

### In-bed cycling

The exercise intervention with in-bed cycling is chosen as it has been shown to be a safe and feasible method to mobilize patients at the intensive care unit (ICU) [24]. For patients who are unable to get out of bed, in-bed cycling could be an alternative and effective method to mobilize patients. The in-bed cycling may further improve endurance and muscle performance of the lower limbs allowing the patient to be able to get out of bed and eventually walking around. As the patient’s physical capacity improves resistance, pace, and exercise time increases in order to induce a continuous training stimulus. Patients allocated to the in-bed cycling groups will daily receive a total of 30 min supervised physical training under the guidance and instruction of an exercise physiologist or physiotherapist student. Patients will exercise for 25 min on the bed bike (Lemco Rehab & Fysio, Helsingør, Denmark) at an individual power, which is set to be tolerated for 25 min and perform exercises from the exercise booklet for 5 min. Again, the selection of exercises is left to the exercise instructor training the patient. Exercises from the exercise booklet is included in the exercise program for the in-bed cycling group in order to introduce and familiarize the patients with the exercises that we encourage them to performed after discharge.

## Training diary and exercise booklet

Upon discharge, patients from the two exercise groups (exercise booklet and in-bed cycling group) will receive a copy of the exercise booklet, a training diary, and encouragement to remain physically active. Patients will be instructed to note information about their physical training at home (e.g., modality, intensity, duration) in the training diary the 1st month after discharge and return the training diary to the study team at the 1-month follow-up visit.

## Follow-up

**Fig. 4:**
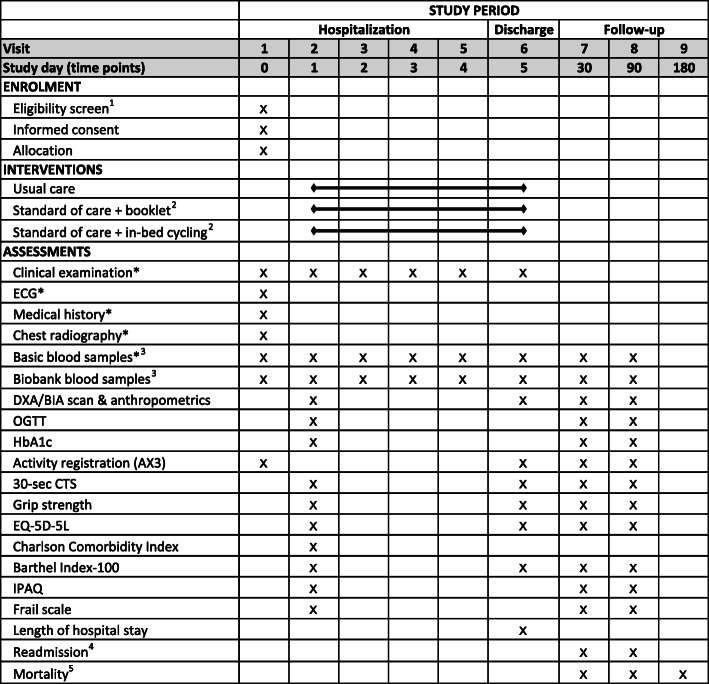
SPIRIT standard protocol items with time schedule of enrolment, interventions and assessments. *BIA* bioelectrical impedance analysis, *DXA* dual-energy X-ray absorptiometry, *ECG* electrocardiogram, *EQ-5D-5L* health-related quality of life, *HbA1c* hemoglobin A1c, *IPAQ* International Physical Activity Questionnaire, *OGTT* oral glucose tolerance test. Asterisk indicates standard usual care. Superscript digit 1 indicates the following: After radiological diagnose of pulmonary infiltrate on chest X-ray/CT in combination with at least one symptom of lower respiratory tract infection at admission, eligibility will be assessed, and potential participants will be approached. Superscript digit 2 indicates the following: The physical training is a supervised exercise program with one session per day. Superscript digit 3 indicates the following: Basic blood samples will be drawn daily as part of routine tests during hospitalization and analyzed for hematologic, renal, endocrine, cardiac, and hepatologic markers. Blood samples stored in the biobank will be analyzed for markers of inflammation. Superscript digit 4 indicates the following: Readmission up to 3 months after discharge. Superscript digit 5 indicates the following: mortality during admission and up to 6 months after discharge. The table is adapted from the SPIRIT recommendations [25]

The follow-up visits take place at the Department of Pulmonary and Infectious Diseases, Nordsjællands Hospital, Hillerød or as home visits, 1 and 3 months after discharge. During the follow-up visits, investigations performed during hospitalization will be repeated (Fig. 4). A phone interview assessing health-related quality of life, physical activity level, functional ability, and frailty will be performed at 6 months after discharge. Data concerning readmission and mortality will be collected from electronic capture from patient files up to 6 months after discharge, while development of type 2 diabetes will be collected up to 2 years after discharge.

## Outcomes and timing of assessments

The attending physician will evaluate whether the patient is ready for discharge. Readiness for discharge is based on the modified Halm’s criteria for clinical stability in CAP [26]. Once the criteria are met, the different measurements scheduled for discharge will be performed. The different outcome variables and timing of assessments are listed in Table 2.

**Table 2:** Outcome

Outcome domain	Specific measurement	Specific metric				Methods of aggregation			Time point
		Value at a time point	Change from baseline	End value	Time to event	Mean	Percent/proportion	Absolute number	
Length of hospital stay	Discharge date–admission date			x		x			Discharge
Readmission	Readmission date–discharge date				x		x	x	3 months
Mortality	Mortality date–discharge date				x		x	x	6 months
Total lean mass	DXA scan		x			x			Discharge
Total fat mass	DXA scan		x			x			Discharge
Total fat-free mass	BIA scan		x			x			Discharge
Leg lean mass	DXA scan		x			x			Discharge
Trunk fat mass	DXA scan		x			x			Discharge
Health-related quality of life	EQ-5D-5L		x			x			Discharge
Activities of daily living	Barthel Index-100		x			x			Discharge
Muscle strength	Hand grip strength		x			x			Discharge
Muscle endurance	30-s chair stand test		x			x			Discharge
Systemic inflammation	Plasma samples		x			x			Discharge
Physical activity	AX3 accelerometers		x			x			After discharge
Glucose metabolism	Oral glucose tolerance test		x			x			3 months
Diabetes status	HbA1c	x					x	x	3 months
Prior physical activity level	IPAQ questionnaire	x					x	x	3 months
Frailty	Frail scale questionnaire	x					x	x	3 months

*BIA* bioelectrical impedance analysis, *DXA* dual-energy x-ray absorptiometry, *EQ-5D-5L* health-related quality of life, *HbA1c* hemoglobin 1Ac, *IPQA* International Physical Activity Questionnaire

### Primary outcome

The primary outcome is length of hospital stay from the time of admission until discharge, with comparison of the groups.

### Secondary outcomes

The secondary outcomes are readmission and mortality measured as time to event within 3 and 6 months from discharge, with comparisons between the groups. Total lean mass, total fat mass, total fat-free mass, leg lean mass, and trunk fat mass measured as mean changes from admission to discharge and from discharge to 3 months after discharge, with comparisons between groups. Health-related quality of life and activities of daily living measured as mean change from admission to discharge and from discharge to 3 months after discharge, with comparisons between groups. Muscle strength and endurance measured as mean change from admission to discharge and from discharge to 3 months after discharge, with comparisons between groups. Systemic inflammation measured as mean change from admission to discharge and from discharge to 3 months after discharge, with comparisons between groups. Accelerometer-based physical activity level measured at admission as mean change from admission to the week after discharge, with comparisons between groups.

### Other outcomes

Additional outcomes are glucose metabolism measured as mean change from admission up to 3 months after discharge, with comparisons between groups. Diabetes status measured as the absolute number of patients with diabetes at 3 months after discharge, with comparisons between groups. Physical activity level and frailty measured at 3 months after discharge (value at a time point), with comparisons between groups.

## Measurement of outcome variables

The time points at which the different outcomes are assessed are depicted in Fig. 4. After inclusion in the study baseline testing is performed (Fig. 4, days 0 or 1). Daily during hospitalization, vital signs are noted, and blood samples are collected (Fig. 4, day 0 until discharge). At discharge, tests performed at baseline are repeated (Fig. 4). Follow-up visits take place 1 and 3 months after discharge, while a phone interview will take place 6 months after discharge (Fig. 4, follow-up).

### Standard usual care

In addition to the assessments required for the trial, patients will undergo examinations (e.g., clinical examinations, CURB-65 score, Early Warning Score (EWS), chest x-rays, 12-lead electrocardiography, and microbiological sampling (sputum, blood cultures, and urine)) prescribed by the attending physician as part of their standard usual care.

### Blood samples

Venous blood samples are collected daily in the morning (between 7 and 9 am) as part of routine tests during admission and will be analyzed the same day according to local guidelines from the Department of Clinical Biochemistry, Denmark, and thus are not affected by the study. This provides access to basic blood work such as complete blood count (white blood cell differential, red blood cell count, platelets), C-reactive protein, coagulation factors, procalcitonine, kidney function, liver function, and electrolytes. In addition to basic blood work diabetes biomarkers such as HbA1c, fasting glucose, fasting insulin, and fasting c-peptide, lipid status (total cholesterol, high-density lipoprotein (HDL) cholesterol, low-density lipoprotein (LDL) cholesterol, and triglycerides) is measured. Besides routine blood samples during admission, additional venous blood samples (plasma, serum, and whole blood) will be collected from all included patients. Plasma and serum samples will be centrifuged at 3,000 × *g* for 15 min at 4 °C and stored together with whole blood in a biobank at − 80 °C until analysis. At the day of inclusion (day 0), plasma, serum, and whole blood samples will be obtained. At days 1–5 and follow-up visits at 1 and 3 months after discharge, plasma and serum samples will be obtained. Biobank samples will be analyzed for inflammatory cytokines and chemokines (e.g., tumor necrosis factor alpha (TNF-α), interleukin (IL)-1β, IL-6, IL-8, IL-10, and IL-18).

### Study questionnaires

At the day of inclusion (day 0), information about prior medical history, socio-demography, comorbidities (Carlson Comorbidity Index (CCI) modified by Quan [27]), smoking, and drinking habits will be collected during an initial interview and supplemented with information from the patient records. Questionnaires concerning health-related quality of life (EQ-5D-5L) [28], ability to perform daily activities (Barthel Index-100) [29], physical activity level (International Physical Activity Questionnaire [IPAQ]) [30], and frailty (FRAIL scale) will be answered in cooperation with the patient at day 1. At discharge from hospital, the questionnaires EQ-5D-5L and Barthel Index-100 will be repeated. At follow-up visits 1 and 3 months after discharge and through phone interview at 6 months after discharge the questionnaires EQ-5D-5L, Barthel Index-100, IPAQ, and Frail scale will be repeated.

### Oral glucose tolerance test

A standard 2-h 75-g oral glucose tolerance test is performed following an overnight fast (8 h). Blood samples are drawn at baseline (0), 30, 60, and 120 min. The blood samples will be analyzed for p-glucose, p-insulin, and c-peptide. As a marker of stimulated insulin sensitivity, the Matsuda index will be calculated as 10,000/√[(baseline glucose × mean glucose) × (baseline insulin × mean insulin)] [31]. As a marker of fasting insulin resistance, Homeostatic Model Assessment (HOMA-IR) will be calculated as HOMA-IR = (fasting glucose × fasting insulin)/22.5 [32].

### Anthropometry and body composition

Weight is measured to the nearest 0.1 kg on an electronic scale (Seca, Hamburg, Germany). Height is measured to the nearest 0.5 cm using a stadiometer. Body mass index (BMI) is calculated as weight (kg)/height (m)^2^. Waist, hip, and arm circumferences are measured to the nearest 0.5 cm. All measurements are performed within the first 48 h of admission (day 0 or 1), at day 5, on discharge, and at follow-up visits at 1 and 3 months. Bioelectrical impedance analysis (BIA) (BioScan touch i8, Maltron International Ltd, UK) is performed at day 0 or 1, at day 5, on discharge, and follow-up visits at 1 and 3 months to assess changes in the total fat-free mass, fat mass, and body fluids/hydration. Dual-energy x-ray absorptiometry (DXA) scans (Prodigy, GE Healthcare, Nederland) are used to assess the regional distribution of lean body mass and fat mass at days 0 or 1, day 5, and follow-up visits at 1 and 3 months.

### Physical activity

IPAQ will be used to obtain information on the level of physical activity prior to hospital admission, ambulatory visits, and phone interview. Posture allocation and physical activity behaviors are measured using 3-axial accelerometer-based physical activity monitors (Axivity AX3, Newcastle, UK). All patients are equipped with accelerometers placed on the right thigh (posture allocation) and on the right side of the lower back (activity intensity) at admission and until discharge (a maximum of 7 days). At discharge and at follow-up visits at 1 and 3 months after discharge, the accelerometers will be re-attached and collect activity data for further 7 days.

### Muscle strength and functional ability

Muscle strength is measured using the handgrip test (SAEHAN DHD-1 Digital Hand Dynamometer, SAEHAN Corporation, South Korea) as an indicator of overall muscle strength, while the 30-s chair stand test (CST) is used to evaluate the functional ability by measuring lower body strength. Both tests are performed at days 0 or 1, day 5, on discharge, and follow-up visits at 1 and 3 months after discharge.

### Strategies for preventing loss to follow-up

As the study has a relatively long follow-up period, different strategies have been incorporated into the study to minimize loss to follow-up. In order to increase patient’s commitment to the study, the research personal is trying to establish a bond with the patient by having the same personal performing tests, instructing exercise sessions, and scheduling follow-up visits. As the patient is being discharged contact information (phone number and email address) is collected. The follow-up visits should be as flexible and convenient for the patient as possible; therefore, the follow-up visits are offered either as hospital or home visits. To minimize absences from planned follow-up visits, patients are reminded about their upcoming visit in the study. After completion of the first follow-up visit, the next visit is scheduled in advanced to reduce the risk of not being able to get hold of the patient.

### Safety

The safety of initiating exercise in patients early during hospitalization is an area of interest. In a newly published systematic review of patients hospitalized with acute respiratory conditions, a total of 17 adverse events were reported across 1146 participants who completed a total of ~ 7420 exercise sessions [33]. Only 1 serious adverse event occurred and resolved spontaneously in the group performing low-intensity exercise [34]. Therefore, there is no clear relationship between the prescription of exercise intensity and adverse events [33]. However, to ensure that our exercise interventions are delivered safely, oxygen saturation and heart rate are continuously monitored during exercise by pulse oximetry (PalmSAT 2500 Series Pulse Oximeter, NONIN Medical Inc., Minneapolis, USA). Any serious adverse events will be reported to the scientific ethics committee of the Capital Region of Denmark which will decide whether any additional precautions should be taken.

## Statistical power and sample size calculations

The sample size calculation is performed using www.powerandsamplesize.com and is based on the primary outcome, length of hospital stay. The expected reduction in length of hospital stay is based on data from a previous study showing a reduction (3.9 days vs. 6.0 days) for patients hospitalized with CAP randomized to either early mobilization or standard usual care [18]. The number of patients with CAP is based on a 2-day difference in length of hospital stay between CAP patients receiving standard usual care compared to standard usual care combined with daily physical training. The standard deviation for this calculation is 3.5 days, as previous observed [18]. Assuming an estimated drop-out rate of 15%, inclusion of 210 patients (70 patients in each group) will provide a power of > 0.8 at a two-sided significance level of 0.025, adjusted for multiple comparisons (0.05/2).

## Challenges and protocol changes due to the coronavirus disease (COVID)-19

Due to the COVID-19 pandemic and a national wide lockdown starting in March 2020, the study was suspended from March 13, 2020, to April 21, 2020. The recruitment process was again suspended from December 7, 2020, to January 4, 2021, due to the second national wide lockdown. After the second lockdown, the steering committee decided to expand the study inclusion to patients with COVID-19 CAP. As COVID-19 patients is isolated during hospitalization, the exercise sessions are limited to the patient rooms, meaning that especially the exercise booklet group is restricted to primarily strengthening exercises. The COVID-19 pandemic has resulted in missed follow-up visits both hospital and home visits as patients have been unwilling to engagement in research-related activities due to the risk of being exposed to COVID-19. Moreover, follow-up visits are performed more often as home visits.

## Statistical analysis and reporting

The results will be reported in accordance with the Consolidated Standards of Reporting Trials (CONSORT) [35]. Sensitivity analysis for missing data will be performed.

### Pre-planned analysis

Available-case analyses will be carried as the main aim is to evaluate the efficacy of the interventions. This means that patients will contribute to the statistical analyses until they drop-out or the study is terminated. Also, as patient drop-out in most cases will be caused by factors, which will happen independently of this study, missing at random will most likely be an acceptable assumption. Additionally, subgroup analyses for patients without COVID-19 CAP will be carried out as patients with COVID-19 CAP may stay longer at the hospital.

For the primary outcome (length of hospital stay), analysis of covariance will be performed to quantify differences between groups. The following covariates will be included in the model: age, gender, CURB-65 score, BMI, and total number of comorbidities (categories 0, 1, 2, > 2). The estimated mean difference (in changes) between the standard usual care group and the two exercise groups (standard usual care vs. exercise booklet and standard usual care vs. in-bed cycling) with the corresponding adjusted confidence interval (CI) and *p* values will be reported. Both CIs and *p* values will be adjusted utilizing correlations between estimates and tests in a less conservative way compared to Bonferroni adjustment [36].

Survival analysis with Kaplan-Meier estimation and Cox proportional hazards regression will be performed to explore differences between groups in time to readmission. Hazard ratios comparing the standard usual care group with the two exercise groups (standard usual care vs. exercise booklet and standard usual care vs. in-bed cycling) will be estimated and reported together with corresponding CIs and *p* values. Kaplan-Meier estimation and Cox proportional hazards regression will be performed to explore differences between groups in time to mortality. Hazard ratios comparing the standard usual care group with the two exercise groups (standard usual care vs. exercise booklet and standard usual care vs. in-bed cycling) will be estimated and reported together with corresponding CIs and *p* values. For readmission, data will be censored 3 months after discharge, while for mortality, censoring will occur 6 months after discharge.

For secondary outcomes (total lean mass, total fat mass, total fat-free mass, leg lean mass, trunk fat mass, EQ-5D-5L score, Barthel Index-100 score, grip strength, 30-s CST, systemic inflammation, and physical activity [AX3]), linear mixed models with treatment by time interaction as a fixed-effects term and patient-specific random intercepts will be fitted to explore differences in changes from baseline between groups. Specifically, the estimated mean difference (in changes) between the standard usual care group and the two exercise groups (standard usual care vs. exercise booklet and standard usual care vs. in-bed cycling) with corresponding CIs and *p* values will be reported. Logarithm transformations will be applied where appropriate (to handle right-skewed distributions), and resulting estimates and CIs will be back-transformed accordingly.

Likewise, for exploratory outcomes (glucose metabolism), linear mixed models with treatment by time interaction as a fixed-effects term and patient-specific random intercepts will be fitted to explore differences in changes from baseline between groups. Specifically, the estimated mean difference (in changes) between the standard usual care group and the two exercise groups (standard usual care vs. exercise booklet and standard usual care vs. in-bed cycling) with corresponding CIs and *p* values will be reported.

For other categorical outcomes (IPAQ score, diabetes status, and frailty score), differences between groups at 3 months will be explored by the chi-squared test (r × c tables) and expressed as counts and percentages, and *p* values will be reported. In all statistical analyses, *p* values < 0.05 will be considered significant.

### Randomization: sequence generation and allocation concealment

Patients are randomly assigned (1:1:1) in permuted blocks according to computer-generated random numbers (R version 3.4.1), to undergo either standard usual care or booklet exercise or in-bed cycling. The randomization is performed at the day of inclusion (within 24 h of admission) after patients have provided informed consent to participate (additional file 2). No stratification factors are used in the randomization algorithm. The randomized sequence is generated centrally by a researcher not involved in the testing. The allocation is delivered to the researcher responsible for the exercise interventions in sealed envelopes at the time of inclusion. Patients are informed about their allocation after baseline assessment (at days 0 or 1) has been completed. The researcher performing the baseline assessment is responsible for informing the patients about their allocation. The study allocation is blinded for the nurses, physiotherapists, and clinicians treating the patients; however, the researcher responsible for the exercise intervention and outcome assessor is unblinded. Even though blinding could limit the occurrence of bias when collecting and assessing data, the researchers involved in this study has no influence on treatment plans or evaluation of readiness for discharge. The risk of bias due to lack of blinding is therefore evaluated to be relatively low.

## Scientific ethics statement

The study is performed according to the Declaration of Helsinki II [37] and is carried out in accordance with the rules in the Danish Act on Processing of Personal Data. All participants receive written and oral information about the study prior to inclusion and their provision of oral and written consent. The participants can always discontinue participation in the study with no obligation to provide a reason.

## Data collection

Prior to study initiation an online Research Electronic Data Capture (REDCap) version 8.10.18 database was built to accommodate patient eligibility and entry of study data for enrolled patients. Data sources include data from patient files and self-reported survey responses and data on anthropometry and functional capacity. All data are entered to our online study REDCap database. Patient identifiable data is stored separately in a secure folder by the investigating center (Department of Pulmonary and Infectious Diseases, Nordsjællands Hospital, Denmark).

To reduce errors in data entry, laboratory results are extracted directly from patient files into data sheets. Similarly, results from BIA and DXA scans are exported directly into data sheets. To secure data quality and completeness, the research group works closely together on a daily basis. Moreover, every second week the project team attend meeting with the steering committee to discuss scientific issues.

## Data monitoring, steering committee, and confidentiality

The data monitoring will be limited to on site, where the project team at the Department of Pulmonary and Infectious Diseases (Nordsjællands Hospital, Hillerød, Denmark) will serve as the data monitoring committee. Monitoring of data will occur as the data is being entered into the online database (REDCap). The project team will ensure that patients is included and treated ethically correct. If adverse events occur, the project team will discuss them with a steering committee before reporting them to the scientific ethics committee of the Capital Region of Denmark, which will ultimately decide if additional precautions must be taken. Moreover, the steering committee is involved in monitoring the overall progress of the trial (e.g., recruitment progress and data collection). The investigators will ensure that the trial is conducted in accordance with the principle of Good Clinical Practice. Confidentiality of the participants is maintained by assigning participants a study number, keeping identifiers separate from data, and storing data a secure database online (REDCap). Scientific reports generated from the study will not contain information that would identify the participants. After the end of the study, all documents will be archived for a period of 15 years.

## Research biobank and future research biobank

A research biobank is created for storing blood samples at the Nordsjællands Hospital, Hillerød. The blood samples are pseudonymized and stored in a freezer in a locked room until analysis. After completion of the study, excess biological material will be transferred to a new biobank for future research within the pneumonia disease. Permission to create a future research biobank will be applied at The Danish Data Protection Agency. The samples will be stored for 15 years in accordance with the current legislation and data protection laws.

## Remuneration/services

No remuneration is paid to the study participants.

## Information about compensation schemes

The study is covered by the patient compensation scheme if, contrary to expectation, damages occur during the study.

## Publication of results

The study protocol adheres to the Standard Protocol Items: Recommendations for Interventional Trials (SPIRIT) [25] (Additional file 1), and the results of this study will be reported according to the CONSORT guidelines [35]. The results from this project will be published in an international peer-reviewed journal regardless of whether they are positive, negative, or inconclusive. If publication in an international scientific peer-reviewed journal is not possible, the results of the study will be published in a report format, which will be made available online. Authorship are based on the Vancouver recommendations: (1) substantial contributions to the conception or design of the work or the acquisition, analysis, or interpretation of data for the work; (2) drafting the work or revising it critically for important intellectual content; (3) final approval of the version to be published; and (4) agreement to be accountable for all aspects of the work in ensuring that questions related to the accuracy or integrity of any part of the work are appropriately investigated and resolved.

## Discussion

Despite the increasing evidence of the adverse consequences related to immobility during hospitalization, patients spend most of their in-hospital time in bed. Although physical activity is a modifiable factor that might prevent hospital-related functional decline, bed rest is deeply rooted in the hospital culture. While mobilization is imbedded in the standardized treatment for patients hospitalized with CAP [21], research has shown that mobilization in daily practice is not viewed as a part of the nurses’ core tasks and is therefore not consistently prioritized [23]. Initiatives that promote physical activity among hospitalized patients are obviously needed. Therefore, we have designed this randomized controlled trial to investigate the effect of supervised physical training among patients hospitalized with CAP compared with standard usual care for CAP. We anticipate the results to provide insight into the importance of being physically activity during hospitalization. Furthermore, we hope to gain valuable insight related to the exercise modalities that can be implemented as part of the standardized treatment for patients hospitalized with CAP. Understanding the mechanism in how physical activity can improve prognosis and diminish the impact of critical illness and hospitalization on mobility and health-related quality of life may inform novel therapeutic approaches in the treatment of patients hospitalized with CAP.

## Trial status

Registration on ClinicalTrials.gov (NCT04094636) started on 1 April 2019. The record was first submitted on 5 September 2019 and posted on 19 September 2019. The recruitment period began on 1 April 2019 and is ongoing. The recruitment is planned to be terminated by March 1, 2022. This protocol is version 2 from October 18, 2018. Corrections in the original study protocol for the prospective cohort study include specifications of the two exercise interventions, sample size calculation, and assessment of functional ability. Important protocol amendments will directly be communicated to all investigators, sponsors, registries, and committees. All items can be found in the protocol.

## Supplementary Information

**Additional file 1.** SPIRIT 2013 Checklist: Recommended items to address in a clinical trial protocol and related documents.

**Additional file 2.** Informed consent and participant information to the large prospective cohort study (in Danish).

## Data Availability

The authors declare that all relevant data will be included in the article and its supplementary files.

## Abbreviations

AX3: 3-axis logging accelerometer
BIA: Bioelectrical impedance analysis
BMI: Body mass index
CAP: Community-acquired pneumonia
CCI: Charlson comorbidity index
CONSORT: Consolidated Standards of Reporting Trials
COPD: Chronic obstructive pulmonary disease
COVID-19: Coronavirus disease 2019
CURB-65: Severity score for CAP
DXA: Dual-energy x-ray absorptiometry
ECG: Electrocardiography
EQ-5D-5L: 5-level EuroQol
HDL: High-density lipoprotein
IL: Interleukin
IPAQ: International Physical Activity Questionnaire
LDL: Low-density lipoprotein
OGTT: Oral glucose tolerance test
REDCap: Research Electronic Data Capture
SPIRIT: Standard Protocol Items: Recommendations for Interventional Trials
TNF-α: Tumor necrosis factor alpha
