# Supplementary material for: The impact of physical training on length of hospital stay and physical function in patients hospitalized with community-acquired pneumonia: protocol for a randomized controlled trial
Source: Trials. 2021 Aug 28;22:571. doi: 10.1186/s13063-021-05503-2 (PMC8397876; doi:10.1186/s13063-021-05503-2)
Supplement: Supplementary file 2 — Additional file 2. Informed consent and participant information to the large prospective cohort study (in Danish). [file 13063_2021_5503_MOESM2_ESM.pdf]

Deltagerinformation om forsøget Surviving Pneumoni (Overlev Lungebetændelse)

Forespørgsel om at deltage i et forskningsprojekt
Vi vil gerne invitere dig til at deltage i et videnskabeligt forsøg om lungebetændelser. Forsøget foregår på Lunge- og Infektionsmedicinsk afdeling på Nordsjællands Hospital under ledelse af den forskningsansvarlige overlæge Birgitte Lindegaard Madsen.

Inden beslutning om deltagelse, skal du forstå formålet og nytte ved forsøget. Derfor er det vigtigt, du læser denne deltagerinformation grundigt.

Ønsker du at deltage, vil du blive inviteret til en samtale om forsøget, hvor denne deltagerinformation uddybes. Der bliver også mulighed for at stille uddybende spørgsmål. Du er velkommen til at tage et familiemedlem, en ven eller en bekendt med til samtalen.

Du skal underskrive en samtykkeerklæring som forudsætningen for at deltage i forsøget. Husk, at du har ret til betænkningstid, før du beslutter, om du vil underskrive samtykkeerklæringen.

Det er frivilligt at deltage i forsøget. Du kan når som helst og uden at give en grund trække dit samtykke tilbage. Deltagelse i forsøget vil ikke få indflydelse på din behandling og pleje under indlæggelsen.

Baggrund og formål
Ambitionen med vores projekt er at forbedre behandlingen af lungebetændelse. Vi har en formodning om, at forstyrrelser i sukkeromsætningen hos patienter med lungebetændelse medfører en dårligere prognose i form af øget dødelighed, længere sygdomsvarighed, dårligere behandlingsrespons, hyppigere genindlæggelser samt ringere livskvalitet efter udskrivelse. Vi har også en formodning om, at ernæringstilstand og fysisk aktivitet før, under og efter udskrivelse har en betydning i forhold prognosen ved lungebetændelse. Derfor vil vi gerne undersøge om fysisk træning og en individualiseret kostplan kan forbedre prognosen. Vi vil også gerne undersøge årsagerne til, hvorfor mange patienter med lungebetændelse udvikler hjertekarsygdomme i forbindelse med og efter infektionen.

Der er fortsat meget, vi ikke ved om lungebetændelse forårsaget af Svær Akut Respiratorisk Syndrom coronavirus 2 (SARS-CoV-2), siden virusset blev opdaget i november 2019. Vi vil gerne inkludere 70 patienter i vores undersøgelse og følge dem over en periode på op til 2 år efter inklusion i studiet. Vi ønsker med den lange opfølgning at undersøge, hvor længe patienter med SARS-CoV-2 udskiller virus og er potentielt smitsomme, og hvorvidt immunforsvaret danner langlivede, beskyttende antistoffer, der forebygger ny smitte med SARS-CoV-2.

Der manglers også viden om, hvordan SARS-CoV-2 påvirker nyrerne. Der er flere internationale undersøgelser, der viser, at patienter svært syge af SARS-CoV har pågående nyreskade. Op mod en fjerdel af patienter, der dør af SARS-CoV-2 lungebetændelse, havde udviklet akut nyresvigt som komplikation til sygdommen. Derfor vil vi gerne undersøge i detaljer, om der er pågående nyreskade under indlæggelse ved at måle biomarkører for nyreskade udskilt i urin over flere dage. Det kan give nye viden om, hvordan man kan forebygge komplikationer i nyrerne hos patienter med svær sygdom pga. SARS-CoV-2.

Projektet er tværfagligt og består både af epidemiologiske undersøgelser, fysiologiske undersøgelser samt laboratorieundersøgelser. Projektet består af 6 delprojekter:

A. Oprettelse af en kohorte af patienter med lungebetændelse, som følges over tid, for at belyse årsager til en dårlig prognose og risikoen for at udvikle diabetes
B. Undersøge, 1) hvor længe patienter med en positiv SARS-CoV-2 test er smitsomme efter udskrivelse, 2) dannelsen af antistoffer mod SARS-CoV-2, og 3) om antistofferne mod SARS-CoV-2 er beskyttende mod ny smitte.
C. Undersøgelse af hvordan nyrerne påvirkes af SARS-CoV-2 under indlæggelse
D. Fysisk aktivitet og ernæringstilstand hos patienter med lungebetændelse
E. Inflammation, immunforsvar, tarmflora og sukkeromsætning hos patienter med lungebetændelse
F. Hjertekarsygdomme hos patienter med lungebetændelse

Inklusionskriterier

- Alder ≥18 år
- Røntgenbillede af lungerne med tegn til lungebetændelse
- Symptomer og/eller kliniske tegn på lungebetændelse (temperatur ≥38.0 °C eller <35.0 °C, hoste med eller uden opspyt, brystsmerter, åndenød eller bilyde ved lungestetoskopi)

Eksklusionskriterier

- Patienter, der Ikke er i stand til at give samtykke, vil ikke blive inkluderet i studiet

Forløbet af forsøget

Ønsker du at deltage i projektet Surviving Pneumonia er det ikke en forudsætning, at du deltager i samtlige delprojekter og får udført samtlige undersøgelser. Du vil få mulighed ved inklusionen i kohorten muligheden for at fravælge de undersøgelser (beskrevet nedenfor), som du ikke ønsker eller kan medvirke i. Vi vil som minimum følge dig over tid ved journalopslag for at følge forløbet af dit aktuelle sygdomsforløb med lungebetændelse samt tilstødende komplikationer som f.eks. hjertekarsygdomme, indlæggelse på intensivafdelingen, genindlæggelser samt udviklingen af diabetes på længere sigt. Derved får vi mulighed for at belyse årsager til en god eller dårlig prognose for patienter indlagt med lungebetændelse.

Ved inklusionen skal du interviewes om bl.a. sociodemografiske forhold, sygehistorie, fysisk aktivitet, livskvalitet, medicinforbrug, rygning og alkoholforbrug. Desuden indhentes informationer om din sygehistorie fra din patientjournal.

I forbindelse med forsøget vil vi foretage undersøgelser af din kropssammensætning, ernæringstilstand, sukkeromsætning og dit hjerte. I løbet af indlæggelsen skal der udføres nogle undersøgelser og indsamles biologisk materiale (blodprøver, afføringsprøver, luftvejsprøver og urinprøver) fra dig til opbevaring i en biobank med henblik på senere laboratorieundersøgelser. Undersøgelserne og prøveindsamlingen vil foregå både under indlæggelse og efter udskrivelsen. Der er planlagt 2 ambulante opfølgningsbesøg inden for 6 måneder efter udskrivelse.

Der er også mulighed for at deltage i et træningsstudie, som er et lodtrækningsforsøg, hvor du enten skal følge et træningsprogram eller være i en kontrolgruppe. Hvis du kommer i træningsgruppen, kommer du til at følge et træningsprogram i samarbejde med en humanfysiolog.

Vi vil undersøge din kropssammensætning med kropsmålinger (højde/vægt/talje- og hoftemål), bioimpendans (måler kroppens fedtprocent og vandindhold) samt DXA-skanning (måler fedtfordelingen samt knogletætheden).

Din fysiske aktivitet skal måles med 2 aktivitetsmålere, som placeres på hhv. højre lår og nedre del af ryggen på højre side (ved hoften) ved indlæggelse indtil udskrivelse. Ved udskrivelse beholder du aktivitetsmålerne på i yderligere 14 dage, hvorefter de returneres med posten. I forbindelse med de 2 planlagte opfølgningsbesøg efter udskrivelse skal du have aktivitetsmålerne på i yderligere 7 dage, hvorefter de returneres med posten. Muskelstyrken vurderes ved måling af håndgrebsstyrke med et hånddynamometer.

I forbindelse med sukkerbelastningstesten skal du drikke 75 g sukker opløst i vand. Der tages blodprøver inden samt 1 og 2 timer efter indtagelsen af sukkeropløsningen for at måle blodsukkeret samt koncentrationen af hormoner som indgår i sukkeromsætningen.

Ultralydsskanning af hjertet foregår ved, at du ligger på din venstre side. Ultralydshovedet påføres gelé og placeres over forskellige områder af venstre del af brystkassen og øvre del af maven for at få de ønskede billeder af hjertet. Selve undersøgelsen varer ca. 20 minutter og er risikofrit.

Biologisk materiale og biobank

Der skal indsamles blodprøver, luftvejsprøver, afføringsprøver og urinprøver fra dig i løbet af forsøget. Der er søgt tilladelse fra Datatilsynet om at opbevare prøverne i 15 år i en biobank på Klinisk Forsknings Enhed på Nordsjællands Hospital.

Luftvejsprøver, afføringsprøver (ca. 10 ml) og urinprøver (10 ml) indsamles ved indlæggelsen bruges til undersøgelse af hhv. forholdene i lungerne, tarmfloraens sammensætning og biprodukter fra stofskifte.

Dagligt under indlæggelsen (til og med 10. indlæggelsesdag) og ved udskrivelsen skal der indsamles 3 blodprøver, i alt 30 ml pr. gang. På indlæggelsesdagen, dag 2, 4, 6, 8, 10 og ved udskrivelsen skal der indsamles 2 blodprøver, i alt 11 ml pr. gang. I forbindelse med hver sukkerbelastningstest indsamles 90 ml blod. Desuden indsamles 20 ml blod til analyse af immunceller uden forudgående opbevaring i biobanken. Dvs. under indlæggelsen indsamles i alt 517 ml blod. Ved opfølgning, der udføres 3 gange inden for 12 måneder efter udskrivelse, skal der også indsamles 5 blodprøver samt blodprøver taget i forbindelse med en sukkerbelastningstest. Dvs. ved opfølgning indsamles 273 ml blod.

Der indsamles i alt 790 ml blod inden for en periode på ~~6~~ 12 måneder, hvoraf 517 ml indsamles under indlæggelse og 273 ml ved opfølgning. Blodprøverne vil senere blive undersøgt for biomarkører for inflammation, hjertets funktion, ernæringstilstanden, sukkeromsætningen, kolesterol og fedtsyrer m.m.

Indsamling af biologiske prøver fra patienter med SARS-CoV-2 er beskrevet nedenfor.

Hvis du ikke længere ønsker at deltage i projektet, har du ret til at få destrueret materialet.

Undersøgelse af patienter med positiv SARS-CoV-2 test
Indsamling af luftvejsprøver og blodprøver til undersøgelse for antistoffer.

Som deltager i denne undersøgelse indebærer det en mere opfattende opfølgning for at indsamle luftvejsprøver og blodprøver. Hvis du ønsker at deltage i denne undersøgelse, kan du ikke deltage i studierne om træning eller ernæring, da det kan indvirke på immunforsvaret. Vi vil indsamle luftvejsprøver (svælg- og næsepodninger) inden for de første 30 dage begyndende på dag 1 (hvor du inkluderes i studiet), 3, 7, 10, 14, 17, 21 og 30. Blodprøver til undersøgelse for antistoffer (4 ml pr. prøve) indsamles samtidig med luftvejsprøverne i løbet af de første 30 dage og 3, 6, 12 18 og 24 måneder efter inklusion i undersøgelsen. Efter udskrivelse fra sygehuset vil en sygeplejerske besøge dig i dit eget hjem for at indsamle luftvejsprøver og blodprøver for at minimere kontakt med sundhedsvæsenet og for nemhedens skyld. Et typisk besøg vil vare 15-30 minutter. Udvikler du symptomer på luftvejsinfektion (f.eks. forkølelse, hoste, halssmerter, feber) i løbet af den toårige opfølgningsperiode, bedes du kontakte projektsygeplejersken, som vil komme på et ekstra besøg for at indsamle et sæt luftvejsprøver og en blodprøve til undersøgelse for hhv. luftvejsvirusser inklusive SARS-CoV-2-virus og antistoffer mod SARS-CoV-2. De prøver, vi indsamler i forbindelse med nye symptomer på luftvejssygdom, er ikke diagnostiske, men skal bruges til at undersøge ny smitte med SARS-CoV-2. Vi foretager ikke kliniske vurderinger af eller tager stilling til håndtering af nye luftvejssymptomer. Derfor skal du kontakte din egen læge eller lægevagten på telefonnummeret 1813 ved behov for lægelig vurdering.

Indsamling af urinprøver
En medicinstuderende vil besøg dig under indlæggelsen på bestemte dage for at indsamle en urinprøve. Hvordan urinen opsamles i praksis, afhænger af om du kan tisse selv eller bruger en urinkateter. Hvis du selv kan tisse, vil den studerende udlevere et prøveglas og hente prøven på et senere tidspunkt. Hvis du bruger et kateter, vil den studerende selv indsamle urinprøven fra kateterposen. Der skal indsamles en urinprøve fra dig på dag 1, 3, 7, 10 og derefter 1 gang ugentligt indtil udskrivelsen samt på udskrivelsesdagen. Urinprøverne skal bruges til analyse af markører for nyreskade.

Praktisk forløb af forsøget

- **Dag 1**
    - Indhentning af informeret samtykke
    - Interview
    - Blodprøve til undersøgelse af immunceller
    - Indsamling af prøver til biobank (3 blodprøver, luftvejsprøver, afføringsprøve, urinprøve)
    - Sukkerbelastningstest (indsamling af blodprøver til biobank)
    - Kropssammensætning (højde/vægt/talje og hoftemål, bioimpedans, og en DXA-skanning) og muskelstyrke (håndstyrke)
    - Vurdering af mundstatus (herunder tænder, mundslimhinde, tunge, tandkød samt synkefunktion)
    - Dysfagitest (vurdering af synkeproblemer)

    - Påbegynd måling af fysisk aktivitet under indlæggelse
    - Ultralydsskanning af hjertet
    - Rejse-sætte sig test
    - Gangtest

- **Dag 2 [indtil udskrivelse]**
    - Inklusion og deltagelse i interventionsstudiet (træning og ernæring)
    - Indsamling af prøver til biobank (3 blodprøver) 1 gang dagligt til og med indlæggelsesdag 10 samt prøver til undersøgelse af blodet størkningsproces (2 blodprøve) dag 2, 4, 6, 8, og 10 under din indlæggelse

- **Udskrivelsesdagen**
    - Blodprøve til undersøgelse af immunceller
    - Indsamling af prøver til biobank (3 blodprøver), undersøgelse af blodet størkningsproces (2 blodprøver) dag 2, 4, 6, 8, og 10 under din indlæggelse
    - Kropssammensætning (højde/vægt/talje og hoftemål, bioimpedans, og en DXA-skanning) og muskelstyrke (håndstyrke)
    - Påbegynd måling af fysisk aktivitet efter udskrivelse med aktivitetsmålere
    - Lungefunktionsundersøgelse
    - Ultralydsskanning af hjertet

- **1. opfølgning (inden for 6 måneder efter udskrivelse)**
    - Interview
    - Indsamling af prøver til biobank (3 blodprøver) samt til undersøgelse af blodet størkningsproces (2 blodprøver)
    - Sukkerbelastningstest (indsamling af blodprøver til biobank)
    - Kropssammensætning (højde/vægt/talje og hoftemål, bioimpedans, og en DXA-skanning) og muskelstyrke (håndstyrke)
    - Ultralydsskanning af hjertet

- **2. opfølgning (inden for 6 måneder efter udskrivelse)**
    - Interview
    - Indsamling af prøver til biobank (3 blodprøver) samt til undersøgelse af blodet størkningsproces (2 blodprøver)
    - Sukkerbelastningstest (indsamling af blodprøver til biobank)
    - Kropssammensætning (højde/vægt/talje og hoftemål, bioimpedans, og en DXA-skanning) og muskelstyrke (håndstyrke)
    - Ultralydsskanning af hjertet

- **3. opfølgning (inden for 12 måneder efter udskrivelse)**
    - Indsamling af prøver til undersøgelse af blodet størkningsproces (2 blodprøver)
    - Undersøgelser og opfølgning hvis du er testet positiv for SARS-CoV-2

- Indsamling af blodprøve til undersøgelse for antistoffer på dag 1, 3, 7, 10, 14, 17, 21 og 30 samt 3, 6, 12, 18 og 24 måneder efter inklusion i studiet
- Indsamling af luftvejsprøver på dag 1, 3, 7, 10, 14, 17, 21 og 30
- Indsamling af urinprøver på dag 3, 7, 10 og derefter 1 gang ugentligt indtil udskrivelse

Forsikringsspørgsmål
Da undersøgelserne foregår på hospitalet både under indlæggelsen og ved opfølgning, er du dækket af Patienterstatningsordningen i tilfælde af skader i forbindelse med deltagelse i forsøget.

Bivirkninger, risici, komplikationer og ulemper
Der er få risici forbundet med studiet. Risikoen ved blodprøvetagning er minimal og er i form af smerter, blødning og infektion.

Der er ingen særlig risiko ved den stråledosis, som man udsættes for i forbindelse en DXA-skanning og svarer til en dags baggrundsstråling.

Der kan være risici ved forsøget, som vi endnu ikke kender. Vi beder dig derfor om at fortælle, hvis du oplever problemer med dit helbred, mens forsøget står på. Hvis vi opdager bivirkninger, som vi ikke allerede har fortalt dig om, vil du naturligvis blive orienteret med det samme. I så fald vil du skulle tage stilling til, om du ønsker at fortsætte i forsøget.

Journaloplysninger
For at gennemføre forsøget er det nødvendigt for forskningsgruppen at indhente oplysninger fra din patientjournal løbende under og efter indlæggelsen. En anden grund til at indhente helbredsoplysninger fra din journal er at overvåge forsøget for at sikre, at deltagelse ikke har en negativ indvirkning på dit helbred.

Håndtering af dine personlige oplysninger
Der oprettes en database, hvor resultater fra forsøget skal indtastes og opbevares. Du vil blive tildelt et pseudonym vha. en omsætningsnøgle, der knytter pseudonymet til dit CPR-nummer, inden persondata indtastes i databasen. Alt indsamlet prøvemateriale vil blive opbevaret under pseudonymet. Omsætningsnøglen samt patientoplysningerne vil blive opbevaret på et sikkert drev på Region Hovedstadens netværk. Projektet vil blive anmeldt til Datatilsynet.

Nytte ved forsøget
Der er umiddelbart ingen direkte nytte for dig ved deltagelse i forsøget. Undersøgelsen tjener at gøre os klogere, hvorved vi kan udvikle og forbedre behandling af lungebetændelser for fremtidige patienter. Vi ønsker med vores projekt at forbedre og skræddersyge behandlingen af fremtidige patienter indlagt med lungebetændelse.

Vi vil vide mere om faktorer, der potentielt kan forbedre overlevelsen, forebygge genindlæggelser samt øge livskvaliteten efter udskrivelse. Målet skal opnås ved en bedre forståelse af patientspecifikke faktorer af

betydning for forløbet af en lungebetændelse, som f.eks. hjertekarsygdomme og andre kroniske sygdomme, ernæringstilstand, fysisk aktivitetsniveau, immunforsvaret og glukoseomsætningen.

Udelukkelse fra og afbrydelse af forsøg

Forsøget afbrydes, hvis deltagelse i forsøget på nogen måde påvirker din behandling og pleje, eller din tilstand forværres i en sådan grad under indlæggelsen, at det ikke er muligt at gennemføre indsamling af de relevante prøver eller undersøgelser.

Oplysninger om økonomiske forhold

Initiativet til og udførelsen af dette forskningsprojekt varetages alene af den til forsøget tilknyttede forskningsgruppe, som ikke har økonomiske interesser i udførelsen eller resultaterne af forsøget. Der vil løbende blive søgt om fondsstøtte fra offentlige og private fonde til betaling af løn, udstyr, laboratorieundersøgelser og materialer mm. Der er allerede opnået støtte fra Augustinusfonden i form af 500.000 kr.

Adgang til forsøgsresultater

Vi bestræber, at projektet skal munde ud i flere videnskabelige arbejder, der skal indgå i afhandlinger og publiceres i videnskabelige tidsskrifter. I tilfælde af fund med umiddelbar klinisk relevans, vil denne viden blive gjort tilgængeligt for klinikkerne gennem udarbejdelse af kliniske retningslinjer. Der er mulighed for at få resultaterne tilsendt, når de foreligger, samt få en invitation til offentlige forsvar af afhandlinger.

Implementering af resultaterne

Såfremt en sammenhæng mellem udvikling af sukkersyge og hjertekarsygdomme hos patienter med lungebetændelser findes, vil vi udvikle nye retningslinjer for opfølgning og udredning af patienter i risiko for at udvikler. Hvis det viser sig, at fysisk træning og individualiseret kostplaner bedre prognosen eller øger livskvaliteten efter udskrivelse, vil vi arbejde på at indføre det som en del af standard behandling.

Vi håber, at du med denne information har fået tilstrækkeligt indblik i, hvad det vil sige at deltage i forsøget, og at du føler dig rustet til at tage beslutningen om din eventuelle deltagelse. Vi beder dig også om at læse det vedlagte materiale "Forsøgspersonens rettigheder i et sundhedsvidenskabeligt forskningsprojekt".

Kontaktperson

Hvis du vil vide mere om forsøget, er du meget velkommen til at kontakte den forsøgsansvarlige læge:

Birgitte Lindegaard Madsen
Forskningsansvarlig overlæge
Nordsjællands Hospital, Lunge- og Infektionsmedicinsk Afdeling
Dyrehavevej 29, Bygning 10, Plan 3, 3400 Hillerød,
E-mail: birgitte.lindegaard.madsen@regionh.dk
Telefonnr.: 48 29 38 74

Med venlig hilsen

Projektet er anmeldt til Videnskabsetiske Komiteer for Region Hovedstaden, anmeldelse nummer: 63765

Informeret samtykke til deltagelse i et sundhedsvidenskabeligt forskningsprojekt.

Forskningsprojektets titel: SURVIVING PNEUMONIA (Overlev Lungebetændelse)

Erklæring fra forsøgspersonen:

Jeg har fået skriftlig og mundtlig information og jeg ved nok om formål, metode, fordele og ulemper til at sige ja til at deltage.

Jeg ved, at det er frivilligt at deltage, og at jeg altid kan trække mit samtykke tilbage uden at miste mine nuværende eller fremtidige rettigheder til behandling.

Jeg giver samtykke til, at deltage i forskningsprojektet og til, at mit biologiske materiale udtages med henblik på opbevaring i en forskningsbiobank. Jeg er indforstået med, at forskningsgruppen tilknyttet projektet løbende vil indhente helbredsoplysninger fra min patientjournal for at gennemføre og overvåge forsøget. Jeg er også indforstået med, at det biologiske materiale skal bruges i fremtidige forskningsprojekter. Jeg har fået en kopi af dette samtykkeark samt en kopi af den skriftlige information om projektet til eget brug.

Forsøgspersonens navn: ____

Dato: _______________ Underskrift: ____

Ønsker du at blive informeret om forskningsprojektets resultat samt eventuelle konsekvenser for dig?:

Ja _____ (sæt x) Nej _____ (sæt x)

Erklæring fra den, der afgiver information:

Jeg erklærer, at forsøgspersonen har modtaget mundtlig og skriftlig information om forsøget.

Efter min overbevisning er der givet tilstrækkelig information til, at der kan træffes beslutning om deltagelse i forsøget.

Navnet på den, der afgiver information:

Dato: _______________ Underskrift: ____

Projektet er anmeldt til Videnskabsetiske Komiteer for Region Hovedstaden, anmeldelsesnummer: 63765
